# ANASFV: a workflow for African swine fever virus whole-genome analysis

**DOI:** 10.1099/mgen.0.001455

**Published:** 2025-09-09

**Authors:** Ke Li, Xu Han, Yanwen Shao, Xinyao Wu, Xiaomin Zhao, Edgar Wayne Johnson‡, Runsheng Li

**Affiliations:** 1 Department of Infectious Diseases and Public Health, Jockey Club College of Veterinary Medicine and Life Sciences, City University of Hong Kong, Hong Kong, PR China; 2 Animal Husbandry & Veterinary Station, Beijing SanYuan Group, Ltd, Beijing, PR China; 3 College of Veterinary Medicine, Northwest A&F University, Yangling, Shaanxi, PR China; 4 Enable Agricultural Technology Consulting, Ltd, Beijing, PR China; 5 Department of Precision Diagnostic and Therapeutic Technology, City University of Hong Kong Matter Science Research Institute (Futian), Shenzhen, PR China; 6 Tung Biomedical Sciences Centre, City University of Hong Kong, Hong Kong, PR China

**Keywords:** African swine fever virus (ASFV), genome assembly, genome polishing, genome QC, nanopore sequencing

## Abstract

African swine fever virus (ASFV) is highly transmissible and can cause up to 100% mortality in pigs. The virus has spread across most regions of Asia and Europe, resulting in the deaths of millions of pigs. A deep understanding of the genetic diversity and evolutionary dynamics of ASFV is necessary to effectively manage outbreaks. Genetic analysis of the ASFV requires sequencing and assembling its genome. Nanopore sequencing is increasingly used for ASFV analysis due to advantages such as long reads and portability. However, applying nanopore sequencing to ASFV genomes faces challenges, notably a higher error rate. Additionally, there is a lack of standardized methods for evaluating genome quality. Furthermore, an increasing number of recombinant isolates between genotypes I and II have been observed, complicating phylogenetic analysis. To overcome these obstacles, we developed ANASFV (analysis of an ASFV whole genome), a comprehensive pipeline that accomplishes four main tasks. First, the pipeline introduces an amplicon sequencing approach that significantly improves genomic coverage, enabling reliable genome assembly, and employs reference-aided polishing techniques to correct small indels caused by nanopore sequencing errors. Second, it establishes a system to provide a relative reference for assessing genome quality in terms of both completeness and accuracy of the assembled genomes. We found that almost all ASFV genomes based solely on nanopore sequencing in the NCBI were of poor quality, which improved significantly after reference-aided polishing. Third, the pipeline introduces a method to rapidly analyse whether an isolate is a recombinant between genotypes I and II, as well as to determine the pattern of recombination based on gene similarity. We identified 11 recombinant ASFV genotypes I and II in the NCBI. Lastly, a comprehensive phylogenetic analysis based on coding sequences was conducted, allowing researchers to generate a refined phylogenetic tree that includes all known ASFV genomes. The ANASFV pipeline facilitates ASFV whole-genome sequencing using the nanopore platform and supports robust downstream bioinformatic analyses by assessing gene completeness and conducting detailed phylogenetic studies based on whole-genome data.

**Impact Statement**

This study presents ANASFV, an integrated workflow that combines amplicon sequencing, reference-based polishing, gene completeness assessment, recombination detection and phylogenetic analysis for African swine fever virus (ASFV). By applying reference-aided polishing, ANASFV effectively corrects small indels in nanopore-derived assemblies, significantly enhancing the quality and accuracy of ASFV genomes – especially those found in public databases. ANASFV introduces a systematic way to evaluate ASFV genome completeness and accuracy, filling a critical gap in assessing the reliability of assemblies generated from nanopore sequencing data. ANASFV enables rapid detection and characterization of recombinant ASFV isolates, particularly those resulting from genotypes I and II. Our study identifies 11 such recombinant isolates from 406 available whole-genome assemblies in the NCBI GenBank database using this function. Using coding sequence-based phylogenetic analysis, ANASFV facilitates the construction of a more accurate and comprehensive evolutionary framework, encompassing all available ASFV genomes and advancing our understanding of the virus’s genetic diversity and evolutionary dynamics.

## Data Summary

Supplementary_figures.docx: Fig. S1 (available in the online Supplementary Material): Gel electrophoresis results of PCR products using primers for 20 kb product. Fig. S2: Gel electrophoresis results of PCR products using primers for 5 kb product. Fig. S3: *Q*-value distribution of reads using primers for 20 kb product. Fig. S4: *Q*-value distribution of reads using primers for 5 kb product. Fig. S5: GC bias of reads from 20 kb primer set and 5 kb primer set. Fig. S6: Frequency of homopolymer occurrence in ASFV and pig genomes. Fig. S7: Frequency of homopolymer occurrence in ASFV and pig genomes with four bases displayed separately. Fig. S8: Recombination plot of 11 recombinant ASFV, a simulated recombinant with 15 times of random recombination event, a genotype I isolate and a genotype II isolate.

Supplementary_tables.xlsx: Table S1: 20 kb primer set. Table S2: 5 kb primer set. Table S3: Completeness evaluation of all available ASFV genomes. Table S4: Statistics of reads from 20 kb and 5 kb primer set. Table S5: Consensus gene list for completeness evaluation. Table S6: Indels in the assembly using only nanopore reads, compared to the assembly (OK358852.1) polished by Illumina reads. Table S7: The long polyG of G_ACD_00350 in 262 genotype II ASFV strains. Table S8: Reference genomes used for polishing 13 low-quality genomes. Table S9: Consensus gene list used for recombination test. Table S10: Homopolished sites of ShanDong2021. Table S11: The statistics of Ka/Ks between ShanDong2021 and ASFV-wbBS01

Supplementary files have been uploaded to the figshare (https://doi.org/10.6084/m9.figshare.27960003).

The ANASFV Python module is available via GitHub (https://github.com/lrslab/anasfv) or PyPI (https://pypi.org/project/ANASFV/). The Newick tree file is also included in the GitHub repo (https://raw.githubusercontent.com/lrslab/anasfv/refs/heads/main/backbone.nwk). We have also provided a Docker container for easier installation. The full document for the pipeline is available at https://anasfv.readthedocs.io/.

The nanopore sequencing reads have been deposited in the Sequence Read Archive (SRA): ShanDong2021 – SRR28789321 (https://www.ncbi.nlm.nih.gov/sra/SRR28789321) (20 kb amplicons) and SRR28789322 (https://www.ncbi.nlm.nih.gov/sra/SRR28789322) (5 kb amplicons); HK_NT_202103 – SRR33967042 (https://www.ncbi.nlm.nih.gov/sra/SRR33967042). The ShanDong2021 genome assembly is available at GenBank: OR660089 (https://www.ncbi.nlm.nih.gov/nuccore/OR660089).

## Introduction

The African swine fever virus (ASFV) is the causative agent of African swine fever (ASF), a severe and often fatal viral disease affecting domestic and wild pigs [1]. Since the initial identification of ASF in Kenya in 1921, the disease has emerged in countries throughout sub-Saharan Africa. The first outbreak of ASFV (genotype I) outside of Africa occurred in the late 1950s, affecting both Europe and South America. In 2007, a new wave of ASFV (genotype II) emerged in Georgia and rapidly expanded to other European countries [2]. In August 2018, ASFV was identified in the northeastern region of China [3]. The ASF outbreak in China had a significant impact due to the country’s substantial share in the global pork market, as China is the largest pork producer. By 2019, ASFV had spread rapidly throughout every region of China [4]. By mid-2019, 13,355 pigs had died from ASFV infection in China, and 1,204,281 pigs were culled to halt the virus’s spread [5]. The virus also extended its reach to other parts of East and Southeast Asia [6,7]. The widespread impact of ASFV underscores the urgent need for global awareness and understanding of the virus.

Oxford Nanopore Technologies (ONT) sequencing offers numerous advantages for sequencing ASFV. ASFV’s genome is ~180 kb in size. Traditional short reads generated by next-generation sequencing (NGS) platforms often struggle to accurately assemble the entire ASFV genome, particularly due to the presence of internal repeat sequences, terminal inverted repeats and genome-end variability. These features can cause significant difficulties in accurate assembly, especially when using short-read sequencing technologies, often resulting in fragmented assemblies, misassemblies or unresolved repetitive regions [8]. In contrast, the long reads produced by ONT sequencing platforms are better suited for generating a high-quality, near-complete ASFV genome. The increased read lengths provided by ONT facilitate the resolution of repetitive regions and improve the detection of structural variations, thereby enabling more accurate and comprehensive genomic reconstructions. As of July 2024, 24 of the 406 ASFV isolates deposited in the NCBI GenBank were assembled with the help of ONT sequencing. Moreover, ONT-based sequencing methods offer a rapid turnaround time compared with NGS [9]. The streamlined workflow, simplified library preparation and real-time data analysis allow for faster generation of whole-genome sequences. Such a quick turnaround is especially important during disease outbreaks or urgent research situations, where timely information is crucial for effective response and control measures.

Several challenges exist in ASFV genome sequencing and assembly. First, substantial host DNA can lead to insufficient ASFV coverage, resulting in a fragmented and incomplete genome assembly. Second, ONT sequencing, especially with the R9.4.1 flow cell, exhibits a higher error rate, leading to numerous small indels [10]. These indels are often found within homopolymers (sequences of consecutive identical bases) due to the difficulty ONT faces in accurately identifying homopolymer lengths. The presence of these indels can lead to fragmented genes, impacting subsequent analyses. In order to solve the high error rate issue in ONT reads, researchers have used hybrid assembly approaches [11,12] or NGS-assistant polishing [13]; however, both strategies depend on supplementary short-read sequencing. Third, there is a lack of a genome quality assessment system after ASFV genome assembly. As a result, some genomes deposited in GenBank may be of low quality.

The recombinant ASFV isolates between genotypes I and II pose a significant threat due to their high lethality and transmissibility among pigs [14]. ASFV has been classified into 24 different genotypes (I to XXIV) based on variations observed in the major capsid protein p72 [15]. Outside of Africa, genotype I and genotype II are the only prevalent genotypes of ASFV. The combination of genetic elements from both genotypes I and II results in a mosaic genome that complicates disease control efforts and vaccine development. The live attenuated vaccines derived from genotype II ASFV have proven ineffective against these recombinant strains [14], underscoring the urgent need for new strategies to combat the recombinant ASFV. Since the first report in 2023, recombinant ASFV isolates have been increasingly detected. As of July 2024, we have identified 11 isolates in NCBI GenBank as recombinants, originating from China, Russia and Vietnam. Recombinant genotypes are a challenge for phylogenetic analysis. Conflicting inferences can arise in the phylogenetic analysis, as different regions of the genome may suggest different evolutionary relationships. The p72 gene (B646L) is currently one of the most widely used genetic markers for ASFV genotyping [16,17]. However, relying solely on p72 can be insufficient for detailed classification, especially within genotype II. Additional loci such as B602L (central variable region) [18,19], intergenic regions [19] and O174L [19] have been shown to provide critical resolution for sub-genotyping and molecular epidemiology. Despite these advancements, determining whether an isolate is recombinant still requires whole-genome sequences, and there remains a lack of convenient tools for detecting recombination events in ASFV.

Constructing phylogenetic trees using the whole coding sequence (CDS) is an ideal method to study the evolutionary relationships among ASFV isolates. The prevalent genotype II isolates in Eurasia show high similarity in the p72 gene. Therefore, differentiating genotype II isolates based solely on the p72 gene is insufficient [20,21]. The presence of numerous repetitive elements in the ASFV genome, along with challenges in genome assembly for certain isolates, complicates the construction of a phylogenetic tree using the full genome [14,22, 23]. Focusing on coding regions for phylogenetic analysis can avoid issues such as poor alignment in repetitive regions and incompleteness of some assemblies [24]. Phylogenetic trees based on protein sequences are better suited for comparing across different species. However, the protein tree is not ideal for studying ASFV isolates since protein sequences are likely to be very similar due to their recent common ancestry. Therefore, the whole CDS tree is an effective solution. Using the whole CDS to build a phylogenetic tree can finely distinguish similar isolates and does not require highly intact genome assemblies. As a result, the utilization of the whole CDS facilitates the incorporation of a greater number of isolates into the tree.

In early 2021, an outbreak of ASF occurred on a farm in Shandong, China, leading to significant livestock mortality. Using the DNA samples collected from the outbreak, we developed and demonstrated our analysis pipeline called ANASFV (Analysis of an ASFV whole genome), which includes sequencing, assembly, polishing, completeness evaluation, recombination test and phylogenetic analysis of ASFV. First, we incorporated PCR amplification with custom-designed primers to ensure adequate coverage and employed reference-guided polishing techniques to effectively rectify small indels introduced by nanopore reads. Second, we developed a system for the completeness evaluation of the ASFV genome. Third, we incorporated a recombination test of the ASFV genome into the pipeline. Lastly, we constructed a phylogenetic tree of 389 ASFV isolates using 188 CDS. We tested this pipeline using the isolate ShanDong2021. As a result, we obtained a high-quality ShanDong2021 genome and a refined ASFV phylogenetic tree.

## Methods

### Sample collection and sequencing

The sample was collected from the lung tissue of sick pigs. Genomic DNA was extracted using the QIAamp MinElute Virus Spin Kit (Qiagen, Germany). Two sets of primers were designed with amplification product lengths of 20 and 5 kb (Tables S1 and S2). The final PCR product of these two sets of primers will miss the first ~94 bp at 5′ end and the last 1.2 kb at 3′ end. The 5 kb set consists of 40 pairs of primers, with an overlap length between amplified products of ~100–500 bp. The 20 kb set consists of ten pairs of primers, with an overlap length between amplified products of ~500–900 bp. The quality and integrity of the amplified DNA products were assessed by agarose gel electrophoresis. The samples were subjected to PCR amplification using the two primer sets, respectively. Subsequently, the amplified DNA products were prepared for sequencing following the nanopore sequencing library preparation protocol (LSK-110, ONT). The library was sequenced in R9.4.1 flow cells (FLO-MIN106D, ONT).

### Basecalling and trimming

The raw nanopore sequencing data were basecalled by Guppy (v5.0.11) with the super-accurate basecalling model. Subsequently, the reads were filtered and trimmed using NanoFilt [25] (v2.8.0), with a minimum average read quality score of 10 and a minimum read length of 1,000 bp. Additionally, the first 50 bases were trimmed using the headcrop option to eliminate potential primer sequences, which are frequently present at the 5′ end of ONT reads and can introduce noise in downstream analysis.

### Genome assembly

The process of selecting the reference genome for mapping assembly involves identifying the genome with the highest number of reads mapped to it. The highest read mapping number reflects a closer genetic relationship between the reads and the reference genome. This approach ensures that the assembly process incorporates the maximum number of relevant reads. The genome exhibiting the highest read mapping number among all available ASFV genomes from the NCBI is regarded as a reference genome (ANASFV: find_near_ref.py). This reference genome was then utilized for mapping assembly of the sequenced data. The alignment was performed using the minimap2 [26] (v2.17) aligner with the -a option and high-quality trimmed reads as input. The alignment results were saved in SAM format. From the SAM file, a consensus sequence was generated using samtools [27] (v1.17). First, the SAM file was converted to the BAM format, excluding unmapped reads (-F 4). The resulting BAM file was then sorted using the samtools sort command. Finally, the consensus sequence was extracted from the sorted BAM file using the consensus command of samtools.

### Polishing the consensus sequence

The consensus sequence was further polished using two rounds of error correction. First, medaka (v1.11.3) (https://github.com/nanoporetech/medaka) was used. The high-quality trimmed reads and the initial consensus sequence were provided as input to the medaka_consensus program. Next, Homopolish [28] (v0.4.1) was utilized to perform additional error correction. We performed blastn on the consensus sequence against the NCBI database, and the closest isolate we obtained was ASFV/pig/China/CAS19-01/2019 (GenBank: MN172368.1). We use this genome as a reference for Homopolish. The Homopolish algorithm used the R9.4.pkl model for correction.

### Phylogenetic analysis

The ASFV genomes used to build a phylogenetic tree were downloaded from the NCBI, covering data up until July 2024 (Table S3). All 188 CDSs from NC_044959.2 (ASFV Georgia 2007/1) were used as a reference as NC_044959.2 (ASFV Georgia 2007/1) is frequently used as a reference in ASFV studies [29,30]. We employed Exonerate [31] (v2.4.0) to fetch the homology gene sequences from each strain’s whole-genome sequence. The CDS of each gene will be used for tree construction. If no hit could be generated by Exonerate with default cutoff, this gene will not participate in the final tree building. The CDS results were then utilized as input for the tree construction of *de novo* mode using uDance [32] (v1.6.3), which employs RAxML (v8) for maximum likelihood tree building. Subsequently, the generated tree served as a backbone for a second iteration of tree construction by adjusting the backbone option to ‘tree’ in uDance, allowing adding the unplaceable nodes during *de novo* tree building. For the total 406 isolates used in our analysis, 16 isolates had CDS sequenced identical with other isolates. One isolate (MT847622.1) could not find its optimal placement in the tree. These 17 isolates were excluded in the final tree. As a result, a total of 389 isolates were shown in the phylogenetic tree. Tree visualization was performed using iTOL [33] (v6.9).

## Results

**Fig. 1.**
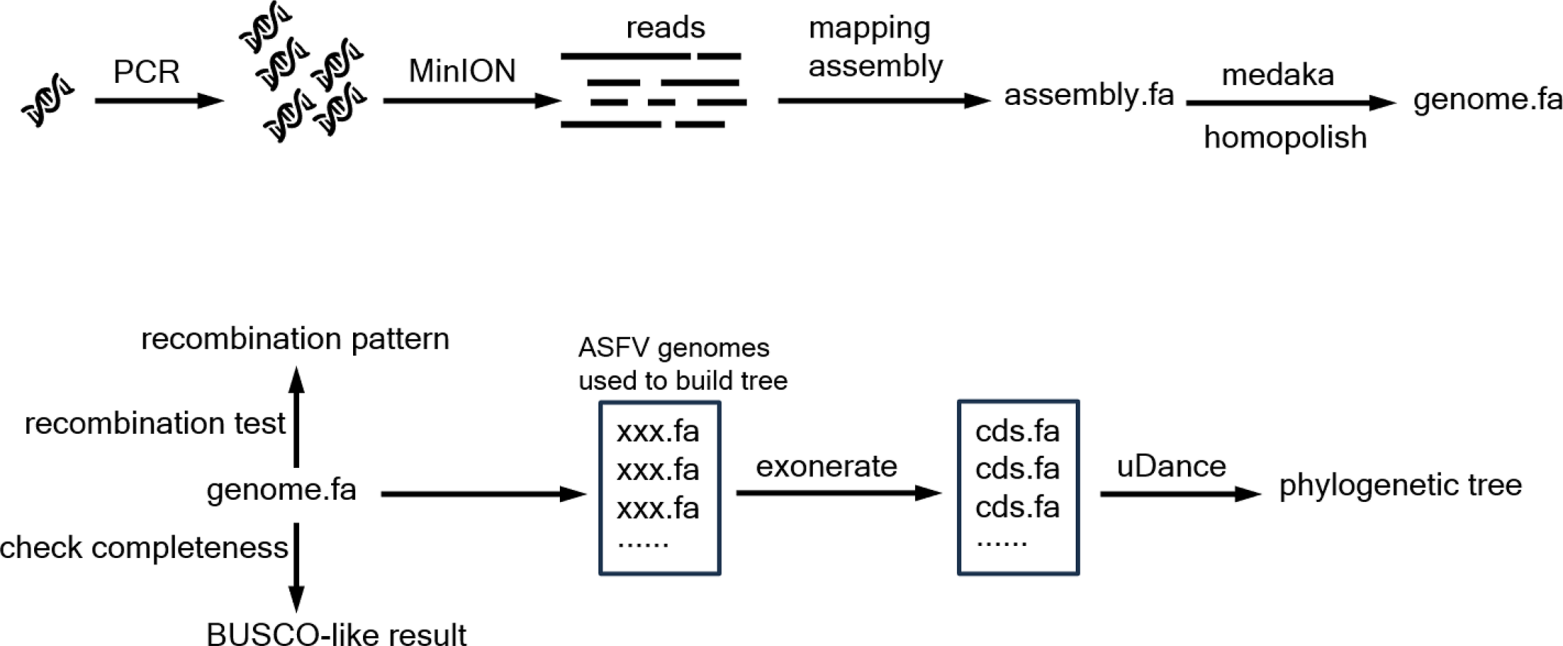
Workflow for ANASFV pipeline.

To overcome the difficulties of nanopore sequencing for ASFV and conduct a more refined phylogenetic analysis of ASFV, we developed a comprehensive pipeline called ‘ANASFV’ that includes sequencing, assembly, polishing, recombination test, completeness evaluation and phylogenetic analysis of the ASFV genome (Fig. 1). We used the DNA sample from the ShanDong2021 isolate to test our pipeline. After performing multiple PCRs, we obtained the amplicon DNA from the ASFV DNA template. Subsequently, we conducted nanopore sequencing using the amplified DNA. The ONT reads were then subjected to mapping assembly to obtain a draft genome. To improve accuracy, we performed two rounds of polishing using medaka and Homopolish. Finally, we obtained the polished genome, which was subjected to completeness evaluation, resulting in a Benchmarking Universal Single-Copy Orthologs (BUSCO)-like evaluation [34]. The ShanDong2021 is a genotype II ASFV and shows no evidence of recombination, according to the recombination test. Additionally, we obtained sequence files for each CDS across genomes of ShanDong2021 and other isolates from the NCBI (Table S3) by employing Exonerate. We then utilized uDance to construct a phylogenetic tree based on the obtained CDS data. The code and the demo data have been deposited on GitHub (https://github.com/lrslab/anasfv). Researchers conducting whole-genome analysis of ASFV using nanopore sequencing can refer to this workflow. For researchers utilizing other sequencing platforms, the downstream analysis of this workflow, such as completeness evaluation, recombination test and phylogenetic analysis, can still be utilized. The ANASFV Python module is available via GitHub (https://github.com/lrslab/anasfv) or PyPI (https://pypi.org/project/ANASFV/). We have also provided a Docker container for easier installation. The full document for the pipeline is available at https://anasfv.readthedocs.io/.

### Effectiveness of the amplicon system

**Fig. 2.**
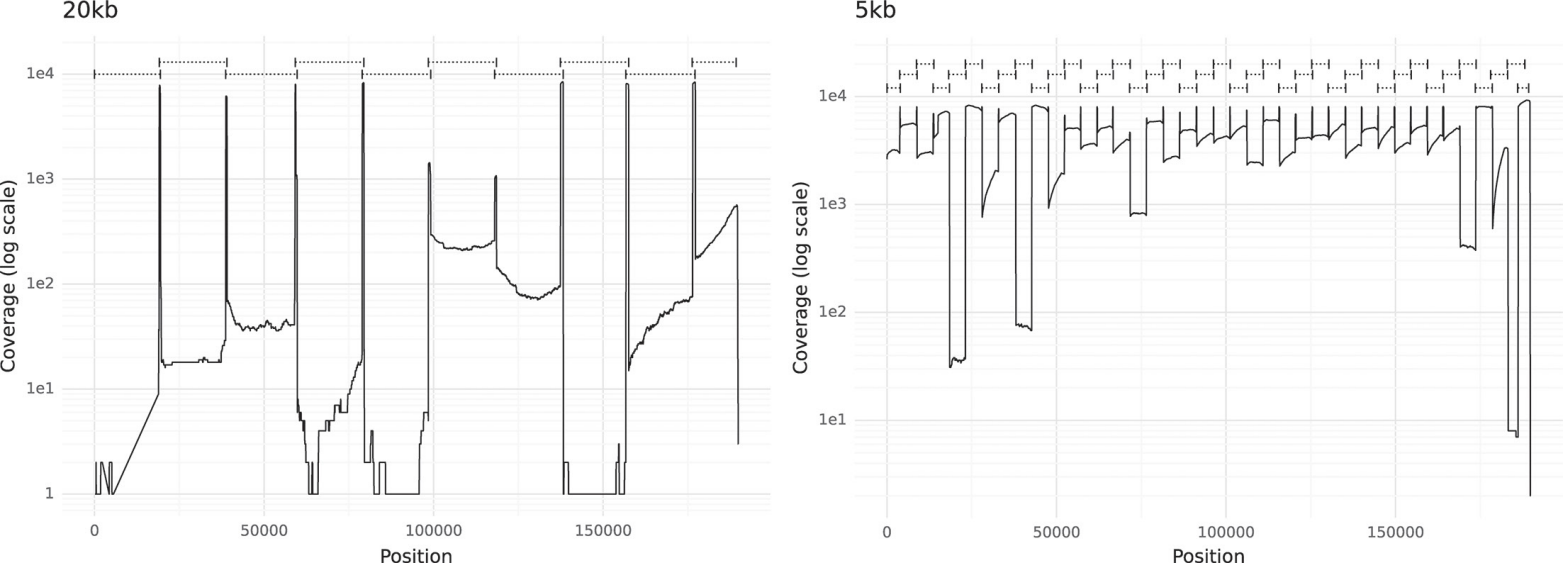
Coverage plot for ASFV amplicons. The logarithmic scale representation of the coverage depth across genomic positions of ShanDong2021, representing the results of amplification products sequencing from primer sets of 5 kb (right) and 20 kb (left), respectively. The positions of primer pairs used are indicated at the top of each figure.

In this ShanDong2021 sample, the 5 kb primer set demonstrated superior performance compared with the 20 kb primer set in terms of amplification efficiency (Fig. 2). Gel electrophoresis of the PCR products (Figs S1 and S2), *Q*-value distribution (Figs S3 and S4) and GC bias (Fig. S5) of the sequencing data from the 20 and 5 kb primer sets is included in the supplementary material. There were four pairs of primers in the 20 kb primer set that exhibited a suboptimal amplification efficiency. These exceptions may have been influenced by factors such as primer design, template quality or other experimental variables. The yield of reads for the 20 and 5 kb primer sets was 748 Mb and 1.6 Gb, respectively (Table S4).

### An evaluation system for the gene completeness of the ASFV genome assembly

Currently, there is no standard method for assessing the completeness of ASFV genomes. BUSCO is a widely used computational tool for assessing the gene completeness of genome assemblies [35]. However, BUSCO is designed for evaluating larger and more complex genomes and is not applicable to ASFV. The ASFV genome is relatively small, and there are significant differences between ASFV genotypes I and II. Similarly, CheckV [36] – optimized for metagenome-assembled viral genomes – is not suitable for ASFV genomes due to its reliance on generic viral databases that do not capture ASFV-specific genomic features. Therefore, we developed a completeness assessment system tailored to the two genotypes of ASFV. We generated consensus sequences for 148 CDSs from genotype II isolates, with 33 multigene family (MGF) genes (MGF_100 : 2, MGF_110 : 3, MGF_300 : 2, MGF_360 : 16 and MGF_505 : 10), and consensus sequences for 149 CDSs from genotype I isolates, with 34 MGF genes (MGF_100 : 2, MGF_110 : 4, MGF_300 : 2, MGF_360 : 16 and MGF_505 : 10). The list of CDS used can be found in Table S5. CDS prediction of ShanDong2021 was performed using Prodigal [37] (v2.6.3), and the predicted CDSs were compared with consensus sequences using blastn (e-value 1e-5). This comparison yielded a BUSCO-like genome completeness evaluation, with C, complete [D, duplicated]; F, fragmented; M, missing; and n, number of genes used. If a consensus CDS term can be mapped to the predicted gene sequences with an identity larger than 90%, and a unique mapping length longer than 90%, the consensus CDS term is considered ‘complete’. A consensus CDS term that cannot find a valid mapping (with an identity greater than 30% and a mapping length greater than 30%) in the predicted genes is considered ‘missing’. Other consensus CDSs with a partial hit are termed ‘fragmented’. The ‘duplicated’ term means that there is more than one ‘complete’ hit in the predicted gene sequences.

### Verification of the effectiveness of reference-aided polish

We used the nanopore data of isolate HK/NT/202103 to verify the effectiveness of reference-aided polishing. HK/NT/202103 has a genome assembled from nanopore R9.4.1 reads and polished with Illumina reads (accession number: OK358852.1, 192,298 bp). We applied the ANASFV mapping assembly pipeline using nanopore R9.4.1 data from HK/NT/202103. Among the ASFV genomes on the NCBI, except for HK/NT/202103 itself (OK358852.1), the genome with the highest read mapping number is BAN20221-4 (PP737708.1). We used BAN20221-4 as a reference and performed mapping assembly to obtain a genome of 187,423 bp. Compared with the previous OK358852.1, this new assembly has some unaligned regions near the two ends, and the new assembly is ~7 kb less than OK358852.1 near the two ends. This OK358852.1 assembly holds highly repetitive sequences at both ends, which could cause misalignment. By excluding the sequences near both ends, we compared the core region from 20 to 190 kb of OK358852.1 to verify the effectiveness of the reference-aided polish in ANASFV.

Compared with the OK358852.1, the raw assembly from nanopore reads before reference-aided polishing had 38 indels (Table S6). The indel number could be further reduced after reference-aided polishing by using ASFV-SY18 (MH766894.3) as a reference, with only five indels remaining (Table S6). The five uncorrected indels were located at genomic sites 20,521, 20,725, 22,526, 104,058 and 184,514, respectively. The three indels at 20,521, 20,725 and 22,526 were all located at long polyG (>11), and Illumina reads also showed variations in the number of Gs inside these regions, indicating that these 3 polyG regions could be variable in length in different viral DNA molecules inside the same ASFV strain. The polyA at position 184,514 had a similar issue. After checking the Illumina reads, we found that the polyA lengths of 4 and 5 could both be supported by NGS reads. The only indel inside a gene region is at 20,725, located at gene G ACD 00350. This gene is not essential, and analysis of all 262 genotype II strains in this study revealed variability in the length of the long polyG of this gene (Table S7). The Illumina reads of HK/NT/202103 also showed reads supporting different lengths of polyG in this region.

### Reference-aided polish helps to resolve the small indels generated by nanopore sequencing

The reads of amplicon sequencing provided sufficient coverage for accurate genome assembly, ensuring that no regions of the reference genomes were left unaddressed. We performed a blastn search of the draft assembly sequence against the NCBI database, and the closest isolate we obtained was ASFV/pig/China/CAS19-01/2019 (MN172368.1). We used this genome as a reference for downstream analysis. An evaluation of quality and accuracy was performed using QUAST (v5.2.0, default parameters) to compare our draft assembly with the reference genome. The evaluation revealed a mismatch rate of 7.98 per 100 kb and an indel rate of 121.75 per 100 kb. After performing reference-aided polishing using Homopolish, the indel rate dropped to 1.6 per 100 kb. Upon comparing the draft assembly with the consensus sequence of genotype II, we obtained the ANASFV completeness evaluation of C: 52.7% [D: 0.0%], F: 45.95%, M: 1.35% and n: 148. After excluding the MGF gene, the completeness evaluation was C: 54.78% [D: 0.0%], F: 43.48%, M: 1.74% and n: 115. Following the application of reference-aided polish using Homopolish, we obtained a completeness evaluation of C: 99.32% [D: 0.0%], F: 0.68%, M: 0.0% and n: 148 and without MGF genes: C: 99.13% [D: 0.0%], F: 0.87%, M: 0.0% and n: 115.

### Completeness evaluation for all ASFV genomes in GenBank

**Fig. 3.**
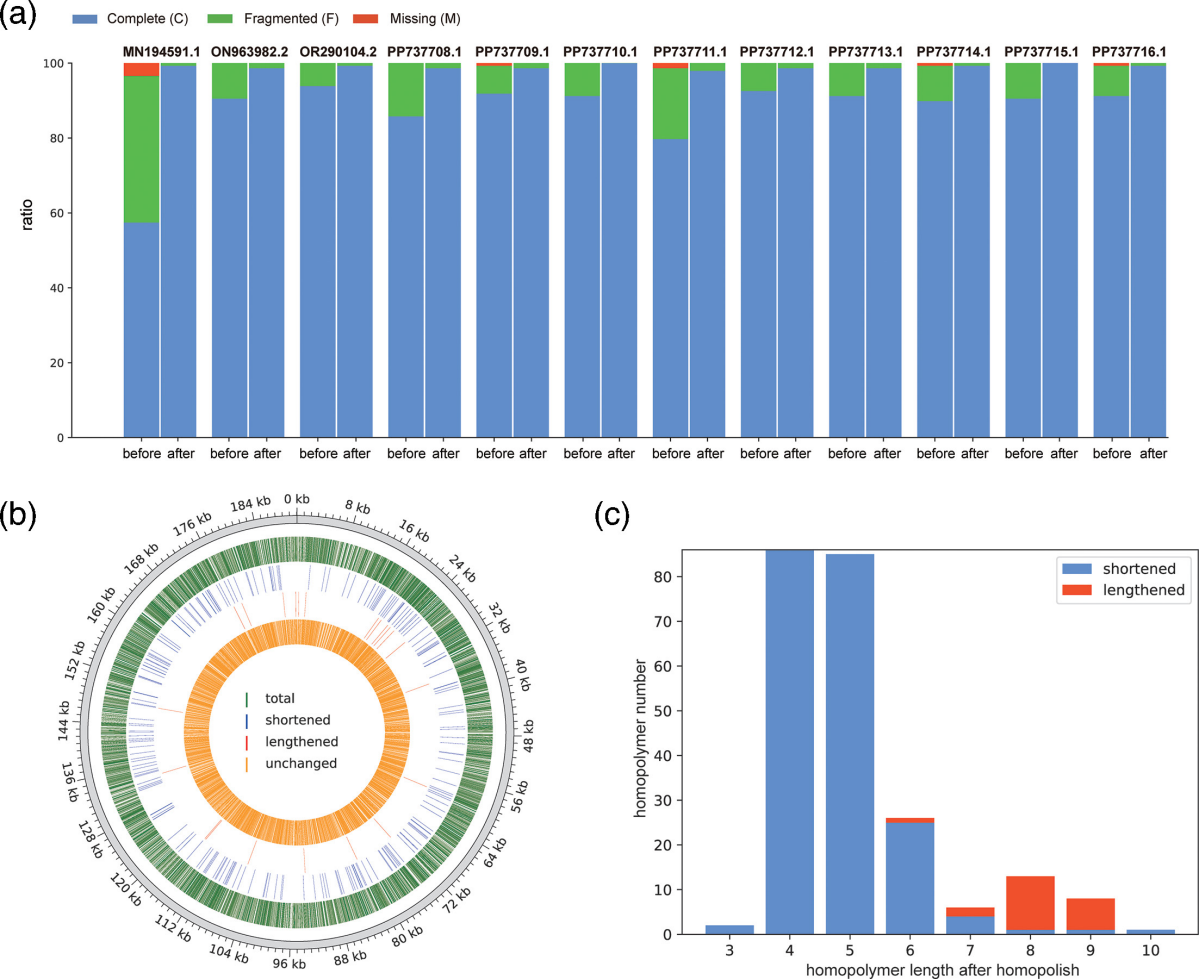
Changes in ASFV genome before and after Homopolish. (**a**) Completeness evaluation of ASFV genomes that were sequenced by nanopore before and after Homopolish. (**b**) The position for revised homopolymers on the ShanDong2021 genome. Only homopolymers with a length greater than three are shown. (**c**) The length distributions for revised homopolymers on ShanDong2021. Only homopolymers with lengths 3–10 are shown. Shortened, the homopolymer length becomes shorter after Homopolish; lengthened, the homopolymer length becomes longer after Homopolish.

We performed the ANASFV completeness evaluation on all available isolates in the NCBI GenBank as of July 2024 (Table S3). Among these isolates, excluding ShanDong2021 (this study), 13 ASFV isolates were sequenced using only ONT sequencing, as shown in Table 1. The completeness evaluation of these 13 isolates indicated poor quality, with completeness scores below 94%. Among the poor-quality genomes, 12 isolates sequenced exhibited issues clearly attributable to insufficient polishing. The genomes of 12 isolates demonstrated inflated gene count and significant gene fragmentation. Some coding regions in the ASFV genome (such as p72, p54 and p30) are closely related to antigenicity. Sequencing errors in these regions can lead to indels, potentially resulting in inaccurate predictions of antigenicity. Among the poor-quality genomes, three isolates had indels in the p72 (B646L) region, resulting in fragmented predictions of this gene. In BAN20221-4, a deletion of a single ‘C’ nt at position 988 of the B646L occurred, resulting in a truncation of the gene. In ASFV/Kyiv/2016/131, a deletion of a single ‘T’ nt at position 1138 of the B646L occurred, resulting in a truncation of the gene at this position. In MSR2022S1, at position 1065 of the B646L, a deletion of a single ‘C’ nt occurred, resulting in a truncation of the gene at this position. We applied reference-aided polishing to the poor-quality genomes using the ANASFV pipeline. The reference genomes used for polishing are listed in Table S8. Gene fragmentations were reduced after polishing (Fig. 3a).

**Table 1.** Nanopore-based GenBank deposited ASFV whole genome sequences before and after polishing

Accession ID	Isolate	Genome size (before/after)	Gene no. (before/after)	Completeness (with MGF) (before/after)	Completeness (without MGF) (before/after)
MN194591.1	ASFV/Kyiv/2016/131	191,911/**191,943**	242/**169**	C: 57.43% [D: 0.0%], F: 39.19%, M: 3.38%, n: 148 **C: 99.32% [D: 0.0%], F: 0.68%, M: 0.0%, n: 148**	C: 51.3% [D: 0.0%], F: 44.35%, M: 4.35%, n: 115 **C: 100.0% [D: 0.0%], F: 0.0%, M: 0.0%, n: 115**
ON963982.2	Philippines A4 2021	192,265/**189,530**	186/**170**	C: 90.54% [D: 0.0%], F: 9.46%, M: 0.0%, n: 148 **C: 98.65% [D: 0.0%], F: 1.35%, M: 0.0%, n: 148**	C: 91.3% [D: 0.0%], F: 8.7%, M: 0.0%, n: 115 **C: 99.13% [D: 0.0%], F: 0.87%, M: 0.0%, n: 115**
OR290104.2	CN/GD/2022	189,416/**189,443**	174/**169**	C: 93.92% [D: 0.0%], F: 6.08%, M: 0.0%, n: 148 **C: 99.32% [D: 0.0%], F: 0.68%, M: 0.0%, n: 148**	C: 93.91% [D: 0.0%], F: 6.09%, M: 0.0%, n: 115 **C: 99.13% [D: 0.0%], F: 0.87%, M: 0.0%, n: 115**
OR449224.1	Ratchaburi_2023_001-MA	213,885/**213,883**	217/**208**	C: 91.89% [D: 16.89%], F: 0.68%, M: 7.43%, n: 148 **C: 91.22% [D: 18.92%], F: 1.35%, M: 7.43%, n: 148**	C: 97.39% [D: 14.78%], F: 0.0%, M: 2.61%, n: 115 **C: 97.39% [D: 14.78%], F: 0.0%, M: 2.61%, n: 115**
OR660089.1	ShanDong2021	190,015/**189,844**	276/**169**	C: 52.7 % [D: 0.0%], F: 45.95%, M: 1.35%, n: 148 **C: 99.32% [D: 0.0%], F: 0.68%, M: 0.0%, n: 148**	C: 54.78% [D: 0.0%], F: 43.48%, M: 1.74%, n: 115 **C: 99.13% [D: 0.0%], F: 0.87%, M: 0.0%, n: 115**
PP737708.1	BAN20221-4	187,609/**187,654**	192/**169**	C: 85.81% [D: 0.0%], F: 14.19%, M: 0.0%, n: 148 **C: 98.65% [D: 0.0%], F: 1.35%, M: 0.0%, n: 148**	C: 86.96% [D: 0.0%], F: 13.04%, M: 0.0%, n: 115 **C: 100.0% [D: 0.0%], F: 0.0%, M: 0.0%, n: 115**
PP737709.1	PAN20211A	189,514/**189,530**	182/**170**	C: 91.89% [D: 0.0%], F: 7.43%, M: 0.68%, n: 148 **C: 98.65% [D: 0.0%], F: 1.35%, M: 0.0%, n: 148**	C: 92.17% [D: 0.0%], F: 6.96%, M: 0.87%, n: 115 **C: 99.13% [D: 0.0%], F: 0.87%, M: 0.0%, n: 115**
PP737710.1	BTG2021KSU1-1	189,540/**189,550**	182/**172**	C: 91.22% [D: 0.0%], F: 8.78%, M: 0.0%, n: 148 **C: 100.0% [D: 0.0%], F: 0.0%, M: 0.0%, n: 148**	C: 91.3% [D: 0.0%], F: 8.7%, M: 0.0%, n: 115 **C: 100.0% [D: 0.0%], F: 0.0%, M: 0.0%, n: 115**
PP737711.1	MSR2022S1	189,514/**189,536**	199/**169**	C: 79.73% [D: 0.0%], F: 18.92%, M: 1.35%, n: 148 **C: 97.97% [D: 0.0%], F: 2.03%, M: 0.0%, n: 148**	C: 78.26% [D: 0.0%], F: 20.0%, M: 1.74%, n: 115 **C: 98.26% [D: 0.0%], F: 1.74%, M: 0.0%, n: 115**
PP737712.1	NEC20230726003	189,537/**189,547**	183/**169**	C: 92.57% [D: 0.0%], F: 7.43%, M: 0.0%, n: 148 **C: 98.65% [D: 0.0%], F: 1.35%, M: 0.0%, n: 148**	C: 93.04% [D: 0.0%], F: 6.96%, M: 0.0%, n: 115 **C: 99.13% [D: 0.0%], F: 0.87%, M: 0.0%, n: 115**
PP737713.1	NEC20230822001	189,528/**189,535**	181/**170**	C: 91.22% [D: 0.0%], F: 8.78%, M: 0.0%, n: 148 **C: 98.65% [D: 0.0%], F: 1.35%, M: 0.0%, n: 148**	C: 91.3% [D: 0.0%], F: 8.7%, M: 0.0%, n: 115 **C: 99.13% [D: 0.0%], F: 0.87%, M: 0.0%, n: 115**
PP737714.1	NEC20230929004A	189,539/**189,543**	180/**169**	C: 89.86% [D: 0.0%], F: 9.46%, M: 0.68%, n: 148 **C: 99.32% [D: 0.0%], F: 0.68%, M: 0.0%, n: 148**	C: 89.57% [D: 0.0%], F: 9.57%, M: 0.87%, n: 115 **C: 99.13% [D: 0.0%], F: 0.87%, M: 0.0%, n: 115**
PP737715.1	NEC20230929004B	189,519/**189,543**	185/**169**	C: 90.54% [D: 0.0%], F: 9.46%, M: 0.0%, n: 148 **C: 100.0% [D: 0.0%], F: 0.0%, M: 0.0%, n: 148**	C: 92.17% [D: 0.0%], F: 7.83%, M: 0.0%, n: 115 **C: 100.0% [D: 0.0%], F: 0.0%, M: 0.0%, n: 115**
PP737716.1	MDR202311F	189,501/**189,514**	183/**170**	C: 91.22% [D: 0.0%], F: 8.11%, M: 0.68%, n: 148 **C: 99.32% [D: 0.0%], F: 0.68%, M: 0.0%, n: 148**	C: 90.43% [D: 0.0%], F: 8.7%, M: 0.87%, n: 115 **C: 99.13% [D: 0.0%], F: 0.87%, M: 0.0%, n: 115**

### Correction of indels in homopolymers in the genome of ShanDong2021

A homopolymer is a sequence of the same nt repeated consecutively, typically when a single nt is repeated more than twice. The ASFV genome contains an excessive number of homopolymers. To better understand the extent and characteristics of homopolymer in ASFV, we conducted a comparative analysis with the pig (Sus scrofa) genome, the host of ASFV, as well as the simulated random genome sequences with the same GC content as the ASFV (38.38%) or pig genome (43.07%). The results show that the frequency of homopolymers longer than 3 nt in the ASFV genome is higher than in the pig genome, and both are higher than in the simulated genomes (Fig. S6). Although the total count of homopolymers in the ASFV genome is higher than in the pig genome, homopolymers composed of ‘A’ and ‘T’ bases occur more frequently in the pig genome compared with ASFV. Conversely, homopolymers composed of ‘G’ and ‘C’ bases occur with a higher frequency in the ASFV genome (Fig. S7). There are 4,866 homopolymers longer than 3 nt in the ShanDong2021 genome. After applying Homopolish, 229 homopolymers underwent changes. Among these 229 changes, 22 were lengthened, while the majority, 207, were shortened (Fig. 3b). In addition to the 229 typical homopolymer changes after polishing, there was also 1 instance where ‘AA’ was changed to ‘A’. Among the indels generated by nanopore sequencing, 90.4% (208 out of 230) were recognized as longer than their actual length and were thus shortened after polishing. Most of the shortened cases (198 out of 208) involved homopolymers ranging from 3 to 6 nt in length. The remaining 9.6% (22 out of 230) indels, which were recognized as shorter than their actual length, consisted of homopolymers ranging from 6 to 9 nt in length (Fig. 3c).

### Description of ShanDong2021

**Fig. 4.**
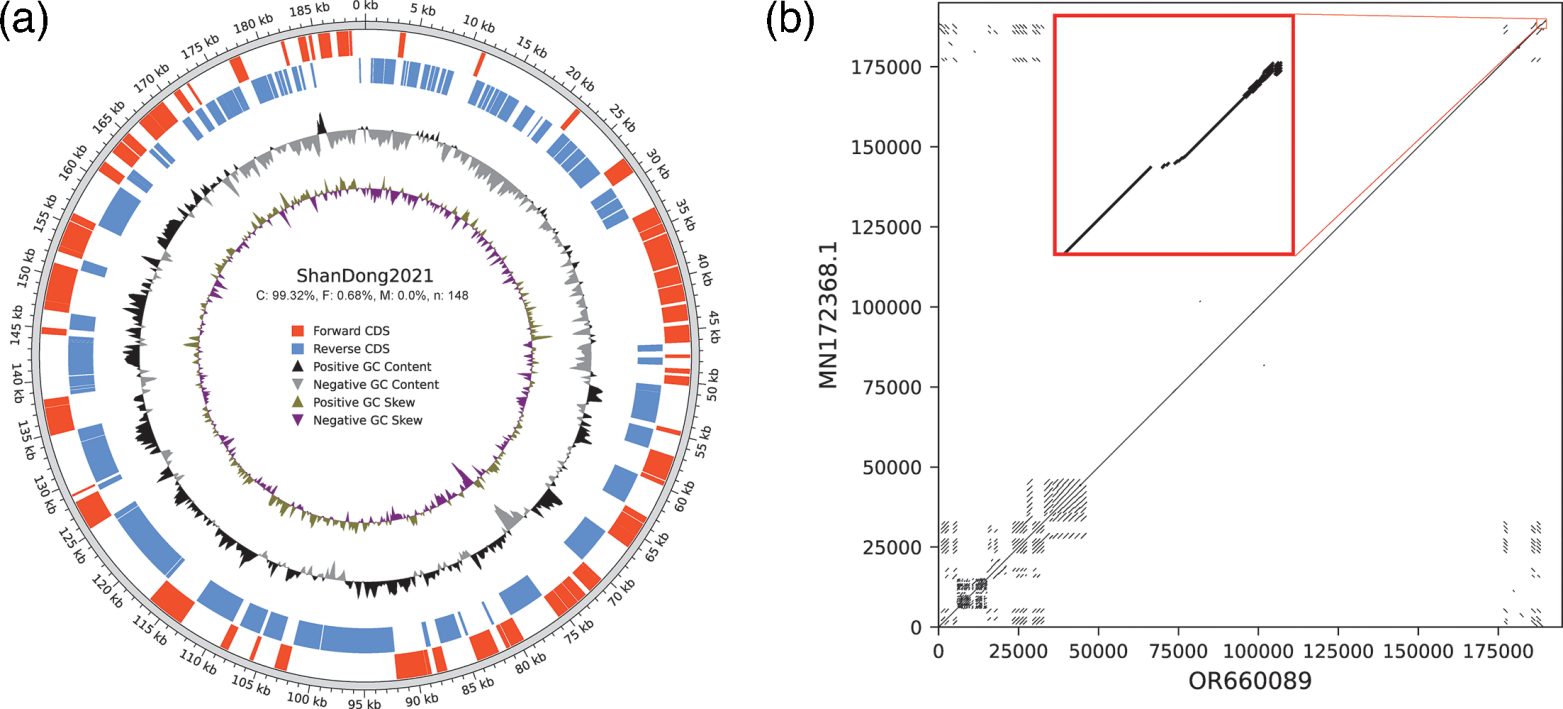
Genomic analysis of ShanDong2021. (**a**) Circular map of ShanDong2021 genome. (**b**) Dot plot of ShanDong2021 (OR660089) to ASFV/pig/China/CAS19-01/2019 (MN172368.1). The red box shows a zoomed-in view of the 187 to 189 kb region.

After polishing, we obtained a final 189 kb ASFV genome assembly of ShanDong2021 (accession ID: OR660089), with 72 genes on the forward strand and 97 genes on the reverse strand (Fig. 4a). In the comparative analysis of the whole genome against a closely related isolate, ASFV/pig/China/CAS19-01/2019 (MN172368.1), ShanDong2021 exhibited an insertion of ~250 bp at the end of MGF_360–21R (Fig. 4b), causing the gene length to increase from 1,071 to 1,092 bp. The dot plot (Fig. 4b) shows that the repeat sequences (mainly MGF genes) are located at both the beginning and the end of the genome, with more at the beginning than at the end.

### A recombination test tool for detecting the recombination between genotypes I and II

**Fig. 5.**
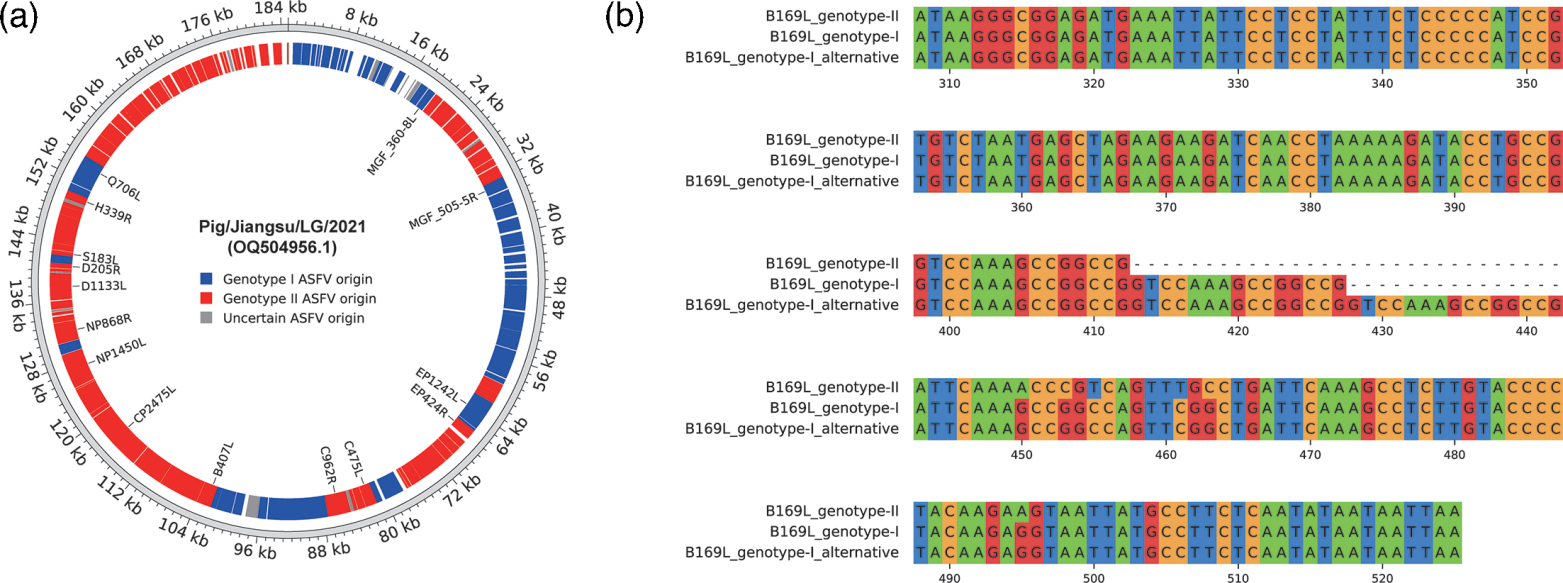
Recombination analysis. (**a**) Recombination plot of a recombinant ASFV isolate named Pig/Jiangsu/LG/2021. The gene names labelled indicate recombination events inside the gene CDS region. (**b**) An alternative B169L of genotype I ASFV compared with B169L gene consensus in genotype I and genotype II.

We generated consensus CDS sets from genotype I and genotype II isolates (Table S9). The CDSs predicted from the ASFV genome using Prodigal were compared with the consensus sequences using blastn with an e-value threshold of 1e-5. Alignments were considered valid if they covered at least 80% of the consensus CDS length. For each CDS, if it is closer to genotype I, it is considered to be from genotype I; if it is closer to genotype II, it is considered to be from genotype II. If there was no blastn hit or the scores for both genotypes were equal, the CDS was marked as uncertain. We found significant differences in the B169L gene among the genotype I isolates (Fig. 5b). Specifically, the ‘GTCCAAAGCCGGCCG’ motif appears once in the B169L gene of genotype II ASFV, twice in tandem repeats in most genotype I ASFV and three times in tandem repeats in an alternative B169L gene found in some genotype I ASFV. Five genotype I isolates (NHV, OURT 88/3, Pig/SD/DY-I/2021, Pig/HeN/ZZ-P1/2021 and Benin 97/1) possess this alternative B169L gene, and all recombinant isolates also carry this alternative B169L gene. Therefore, to more accurately identify the gene, we included the alternative B169L in the consensus CDS set of genotype I.

### Recombination test for all ASFV genomes in GenBank

We performed the recombination test on all available ASFV isolates in the NCBI GenBank as of July 2024. Eleven isolates were identified as recombinants of genotypes I and II (Table 2). As an example, the recombination patterns of the recombinant isolate Pig/Jiangsu/LG/2021 (accession ID: OQ504956.1) are shown (Fig. 5a). The existing ASFV recombinants exhibit one sub-gene recombination event. Since our method does not involve the sub-gene level, the recombinations within genes are not reflected in the figure. The recombination patterns of all 11 recombinant isolates were consistent (Fig. S8). In order to verify the reliability of the recombination test. We have randomly generated one recombinant strain from the fusion of genotype I sequences (NC_044957.1) and genotype II sequences (NC_044959.2) with 15 times of recombination event. The code used to generate the fusion has been included in ANASFV (recombinant_sim.py). All the 15 recombination events could be detected using ANASFV (Fig. S8).

**Table 2.** Isolates that were identified as recombinants of genotypes I and II by ANASFV recombination test

Accession ID	Isolate	Country
OQ504954.1	Pig/Henan/123014/2022	China
OQ504955.1	Pig/Inner Mongolia/DQDM/2022	China
OQ504956.1	Pig/Jiangsu/LG/2021	China
PP348677.1	Primorsky 2023 DP-4560.Rec	Russia
PP478517.1	NJS23-1	China
PP478518.1	NJS23-2	China
PP712068.1	JX23-01	China
PP712069.1	JX23-02	China
PQ010732.1	rASF12-avac02	Vietnam
PQ010733.1	rASF1/2-avac03	Vietnam
PQ010734.1	rASF1/2-avac07	Vietnam

### Phylogenetic analysis

**Fig. 6.**
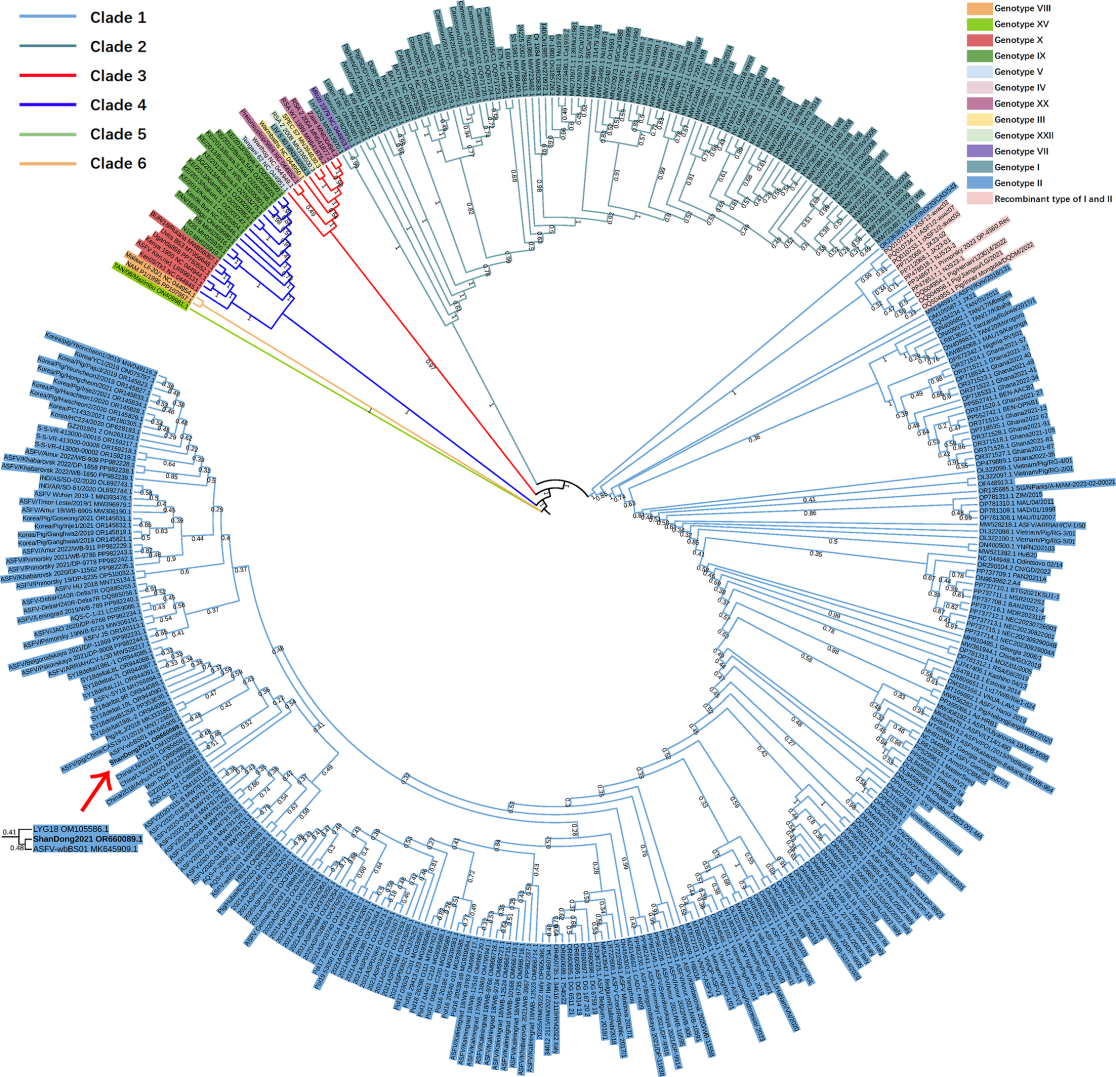
Phylogenetic analysis with 188 CDSs from 406 available ASFV whole genomes deposited in GenBank (July 2024). The ShanDong2021 isolate is highlighted using bold letters and a red arrow. A magnified image of the branch of ShanDong2021 is shown in the lower right corner. Isolates are represented using their NCBI nt accession numbers and their isolate names. The colours of the branches indicate the six clades identified by our study. The background colour of the isolate name indicates different genotypes.

We used all available ASFV genomes from the NCBI GenBank as of July 2024 (Table S3) to construct the phylogenetic tree, including 12 genotypes and the recombinant type I-II (Fig. 6). Six distinct clades can be identified. Isolates from genotypes V, XX, IV, III and XXII, which are considered to be different genotypes, are grouped together in Clade 3. Clade 3 also contains LIV_5/40 [38], an isolate considered to be of genotype I. This mixed clade has been observed previously [29,39, 40]. Clade 3 is consistent with that of the epsilon clade [39] and Biotype 3 [29] in previous studies. Clade 3 may be partly explained by previously reported concerns regarding the quality of several ASFV genome assemblies, including LIV_5/40, SPEC57, Zaire, RSA2004/2, RSA2008/2 and RSA1999W/1 [41]. These assemblies may contain a high number of sequencing or assembly errors, potentially affecting phylogenetic placement and contributing to the formation of atypical clades. Moreover, several isolates – such as Warmbaths (III), Tengani 62 (V) and Pretoriuskop/96/4 (XX) – have been reclassified into a proposed new ‘genotype 2’ based on their identical p72 protein sequences [41]. Our findings support this reclassification, as these isolates grouped together in Clade 3. There are 256 genotype II and 89 genotype I isolates in the tree. Eleven recombinants are located at the root of Clade 1. It is important to note that the placement of recombinant strains does not reflect their true evolutionary history due to the mosaic nature of their genomes. Moreover, genome completeness can also introduce biases in phylogenetic inference. Specifically, some low-quality genomes – often characterized by missing or fragmented CDS – tend to cluster near the root, such as ASF/IND/20/CAD/543 (completeness: C: 85.14% [D: 0.0%], F: 3.38%, M: 11.49%, n: 148) at the root of Clade 1. This basal placement may not reflect true evolutionary divergence, but rather artefacts caused by incomplete gene representation. The ShanDong2021 isolate was clustered with LYG18 (OM105586) and ASFV-wbBS01 (MK645909), both of which were sampled from China. ShanDong2021 and ASFV-wbBS01 formed a small clade, indicating a close evolutionary distance between the two. Three genes, I196L (Ka/Ks: 0.24238), M1249L (Ka/Ks: 0.335183) and MGF_360–21R (Ka/Ks: 0.436954), have exhibited significant divergence between the two genomes, suggesting that these genes might be under stronger positive selection.

## Discussion

The short-read length of NGS poses challenges for genome assembly, particularly in resolving repetitive regions such as those found at both ends of the ASFV genome. In contrast, ONT sequencing can generate ultra-long reads, which are capable of spanning most repetitive regions, resulting in assemblies that more accurately reflect the true genomic structure. The feasibility and effectiveness of amplicon sequencing for the ASFV genome, using primers targeting 10 kb amplicons, have been supported by a previous study [42]. In our study, the primer set of 5 kb amplicon outperformed the primer set of 20 kb amplicon. However, this preference is not absolute, as the amplification efficiency depends on the quality of the DNA. For specific needs, researchers can utilize primerdiffer [43] to design their own primer set accordingly. Due to uneven amplification efficiency across different genomic regions, the resulting coverage was highly variable. In addition, the overlapping regions between adjacent PCR products were often limited. These characteristics differ significantly from whole-genome sequencing data, for which tools like Flye and Canu are optimal. As a result, *de novo* assembly produced fragmented assemblies with multiple contigs, which were insufficient for downstream analysis. Given these constraints, we adopted a reference-guided assembly approach, which allowed us to reconstruct genomes efficiently.

The development of the ANASFV completeness evaluation system for the ASFV genome has provided a valuable tool to assess the gene completeness of genome assemblies. BUSCO has become an essential genomic tool for genome completeness evaluation that uses recent data from many species and has broader applicability than the popular but now discontinued Core Eukaryotic Gene Mapping Method (CEGMA) [44]. However, BUSCO is not applicable to viral genomes. The genome of ASFV is ~170 to 200 kb in size; it is a very large viral genome which requires quality assessment. And we demonstrated that a BUSCO-like genome assessment method can be applied to ASFV. Since there are relatively few available genomes for other ASFV genotypes, we only established consensus gene sets for genotype I and genotype II. Therefore, the ANASFV completeness evaluation is more effective for the currently prevalent genotype I and genotype II. As more complete genomes become available for additional ASFV genotypes, the system can be expanded to the broader diversity of ASFV. It is worth noting that low gene completeness does not necessarily mean low genome quality. For example, Ba71v is a nonpathogenic strain derived from WT strain Ba71 [45]. Ba71v has losses and changes of genetic information compared with WT strain. Therefore, Ba71v has a lower score in the completeness evaluation (Table S3). The ANASFV completeness evaluation pipeline fills a gap in the tools available, allowing researchers to conveniently assess the quality of the ASFV genome assemblies.

In the genome polishing process, we employed reference-aided polishing, which has shown remarkable effectiveness in reducing errors introduced during ONT sequencing. ONT sequencing offers the advantage of long reads, which are valuable for resolving complex genomic structures; however, ONT data are known to have a higher base-level error rate compared with short-read technologies. In particular, ONT is prone to sequencing artefacts in homopolymeric and GC-rich regions, often resulting in indels. To address these errors, the common method is to polish with high-quality short reads of NGS. There are many tools for this kind of polish, such as HyPo [46], NextPolish [47], ntEdit [48], POLCA [49] and Polypolish [50]. However, sometimes, researchers only have ONT reads. Currently, most of the ASFV genomes deposited in the NCBI GenBank that are solely based on ONT sequencing have the issue of insufficient polishing. The tool we used, Homopolish, focuses exclusively on correcting homopolymer-associated indels based on a reference and does not alter mismatches, non-homopolymer-associated indels or structural variations, including those in MGFs or terminal repeat regions. As such, the risk of overwriting true sequence diversity is minimal with this targeted approach. The results obtained from applying the completeness evaluation system to the nanopore-based ASFV genomes demonstrated the effectiveness of reference-aided polishing in improving ONT sequencing errors in the absence of NGS reads polishing. For researchers who only use ONT sequencing to obtain the ASFV genome, it is beneficial to employ reference-aided polishing to address indel errors that may persist due to insufficient polishing.

In our study, uDance was used to construct the whole CDS tree [32]. uDance implements a divide-and-conquer strategy that simplifies the phylogenetic tree construction process. By utilizing parallel processing, uDance significantly reduces the time needed for tree construction. A core CDS list may be more accurate in inferring the relationship between different genotypes [51]. However, in order to distinguish genotype II strains with higher resolution, we currently would still suggest using 188 CDS from Georgia 2007/1 as a reference. The ANASFV pipeline automates the process of downloading all available ASFV genomes from the NCBI database, performing CDS prediction and alignment. For researchers not starting from scratch, we also provide our tree file (Newick format), which can serve as a backbone tree to add new isolates by adjusting the backbone option to ‘tree’ in uDance.

In conclusion, our study successfully demonstrates the application of ONT sequencing coupled with custom-designed PCR primers for the genome sequencing of the ShanDong2021 ASFV isolate. The use of Homopolish significantly enhanced the accuracy of the assembled genome. Additionally, the development of a gene completeness assessment system and a recombination test provided valuable tools for evaluating assembled genomes and detecting recombinant isolates. The construction of a comprehensive phylogenetic tree offered deep insights into the genetic characteristics and evolutionary relationships of ASFV isolates.

Since the inception of our study in December 2023, the number of ASFV genomes sequenced using only ONT technology has increased from 4 to 14, and the number of recombinant ASFV genomes available in the NCBI has risen from 3 to 11 by July 2024. This trend indicates a growing adoption of ONT sequencing in ASFV research and an increase in recombinant ASFV isolates. The ANASFV pipeline we developed is well-positioned to support this expanding field, providing researchers with a robust workflow for ASFV whole-genome analysis. By facilitating accurate genome assembly, quality assessment, recombination detection and phylogenetic analysis, the ANASFV pipeline contributes significantly to the understanding and control of ASFV, ultimately aiding efforts to mitigate the impact of this devastating virus on the pig industry.

## Supplementary material

10.1099/mgen.0.001455 Uncited Supplementary Material 1.

10.1099/mgen.0.001455 Uncited Supplementary Material 2.

## Abbreviations

ASF: African swine fever
ASFV: African swine fever virus
BUSCO: Benchmarking Universal Single-Copy Orthologs
CDS: coding sequence
MGF: multigene family
NGS: next-generation sequencing
ONT: Oxford Nanopore Technologies
