# Supplementary material for: ANASFV: a workflow for African swine fever virus whole-genome analysis
Source: Microb Genom. 2025 Sep 9;11(9):001455. doi: 10.1099/mgen.0.001455 (PMC12452174; doi:10.1099/mgen.0.001455)
Supplement: Uncited Supplementary Material 1. [file mgen-11-01455-s001.pdf]

Supplementary Figures

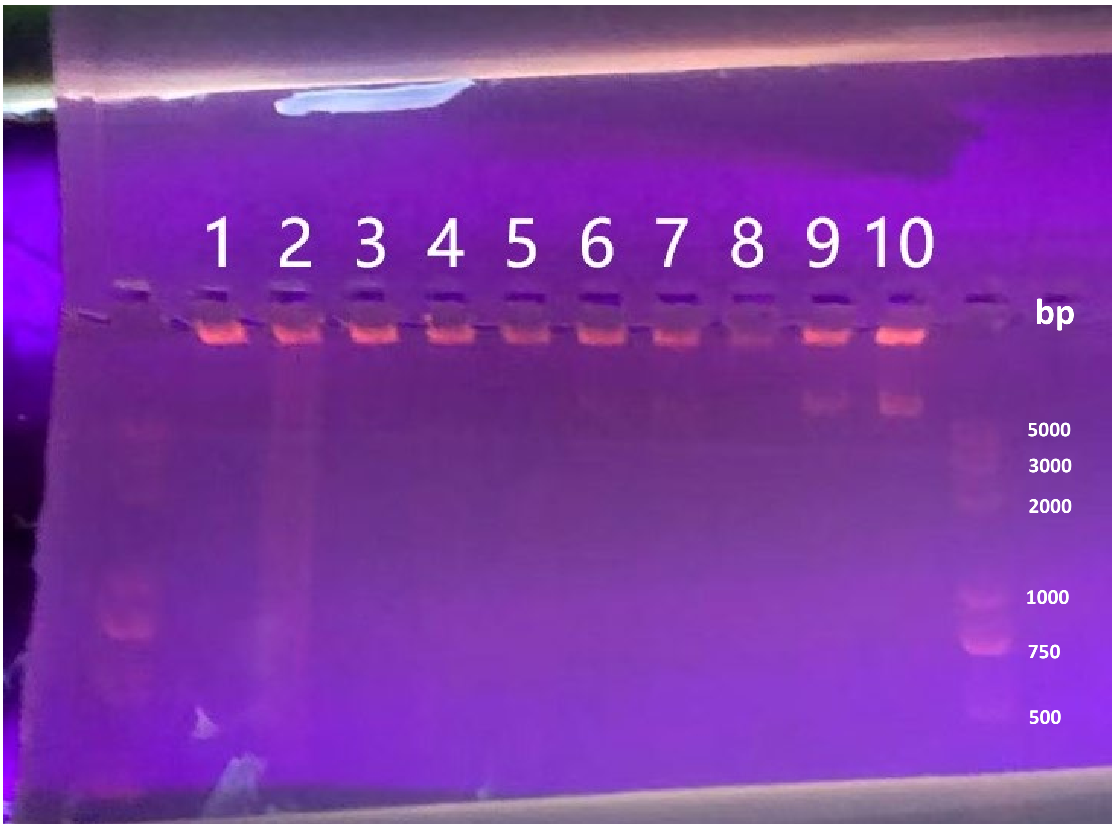

Figure S1. Gel electrophoresis results of PCR products using primers for 20 Kb product. The grouping of primer pairs is shown in Table S1.

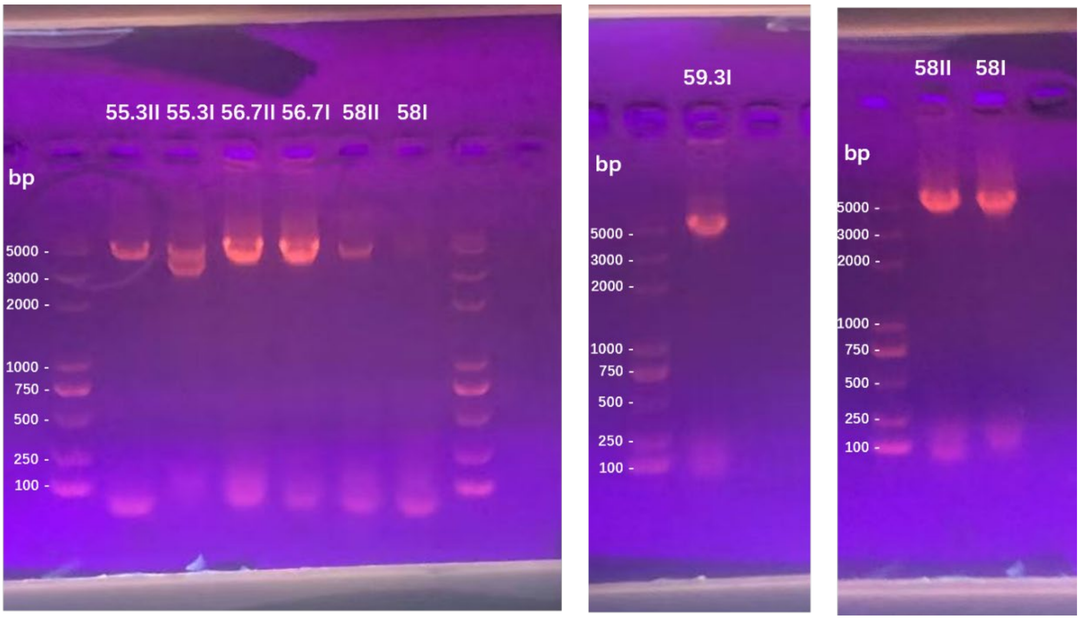

Figure S2. Gel electrophoresis results of PCR products using primers for 5 Kb product. The primer pairs were subjected to multiplex PCR, with primer pairs with similar annealing temperatures combined into single reactions. The grouping of primer pairs is shown in Table S2.

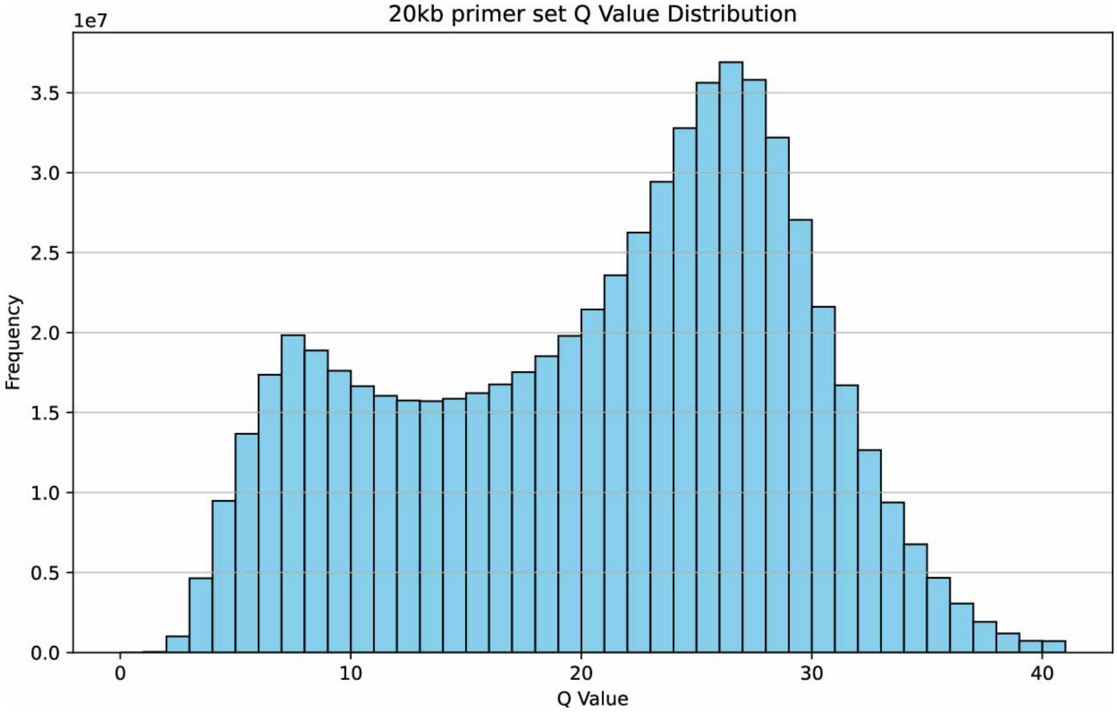

Figure S3. Q value distribution of reads using primers for 20 Kb product.

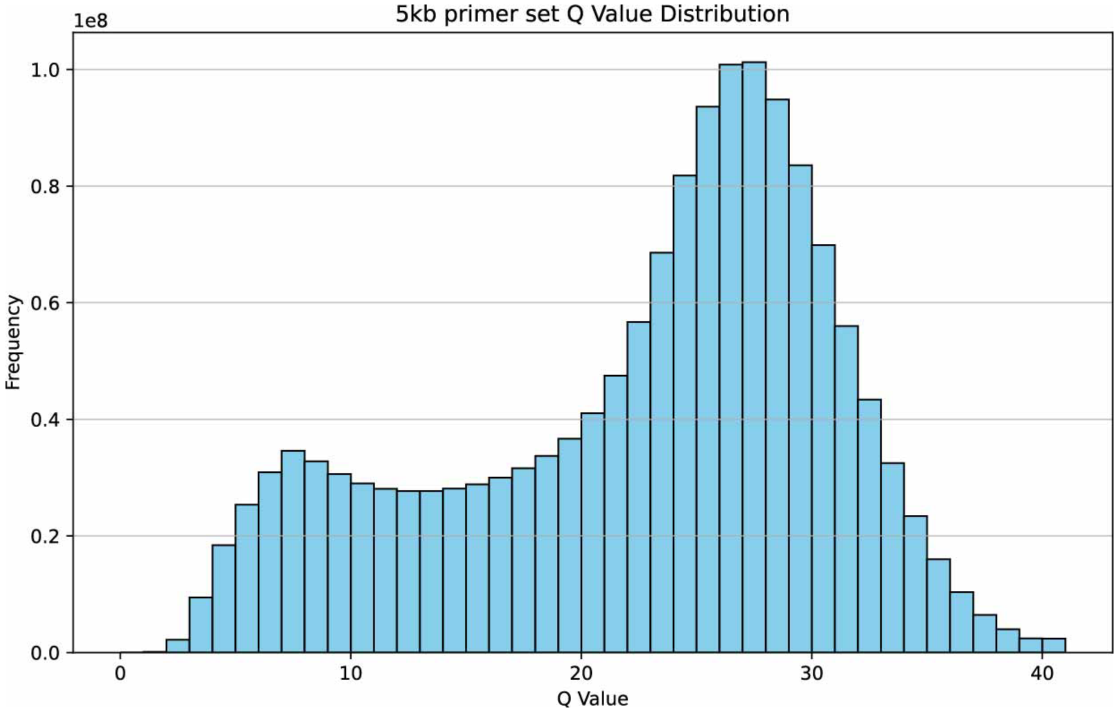

Figure S4. Q value distribution of reads using primers for 5 Kb product.

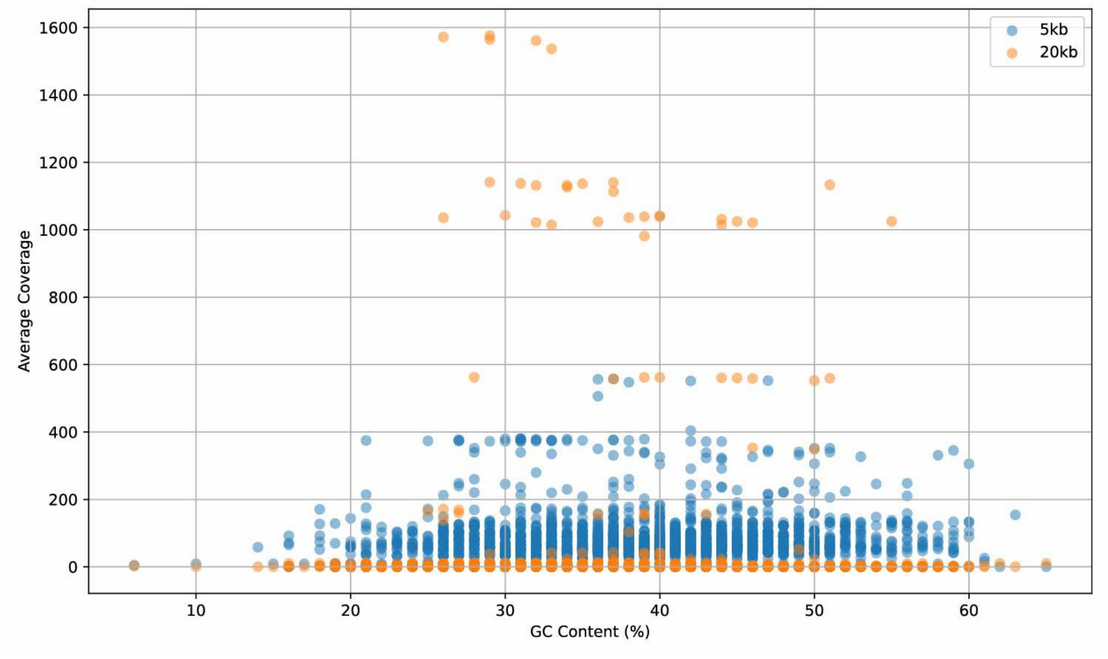

Figure S5. GC bias of reads from 20 Kb primer set and 5 Kb primer set (bin=100bp).

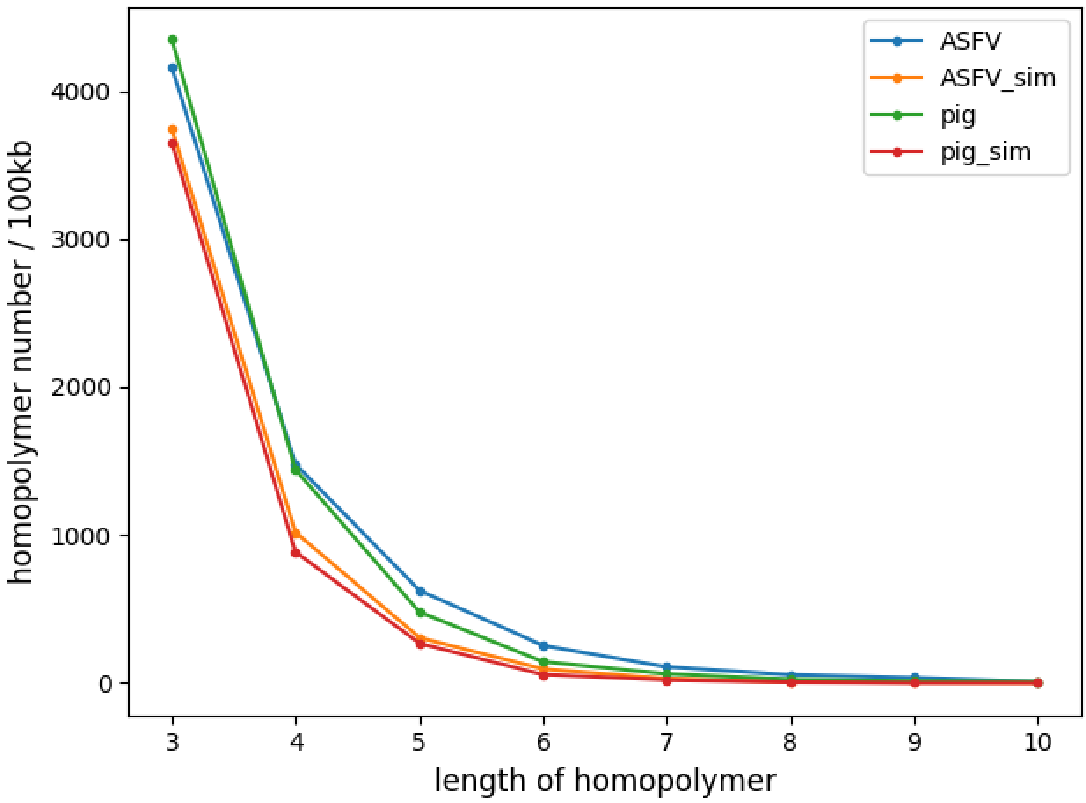

Figure S6. Frequency of homopolymer occurrence in ASFV and pig genomes. "sim", simulated genome using the same GC content.

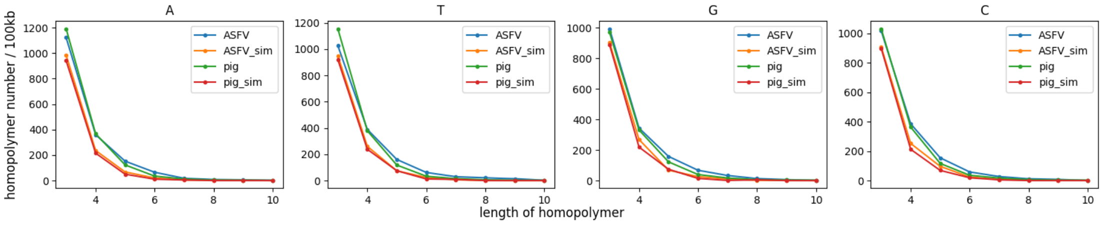

Figure S7. Frequency of homopolymer occurrence in ASFV and pig genomes. The four bases are displayed separately. "sim", simulated genome using the same GC content.

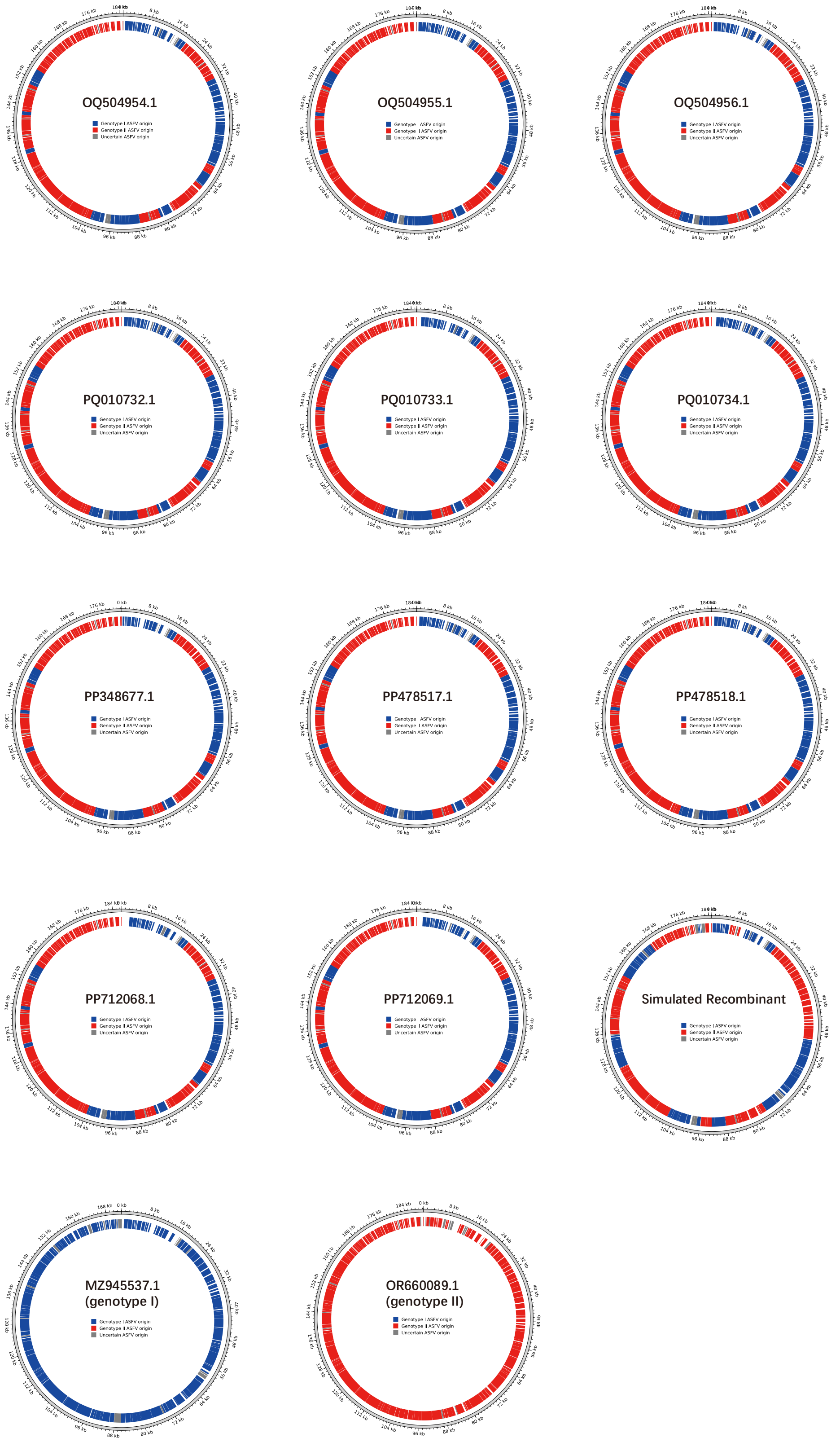

Figure S8. Recombination plot of 11 recombinant ASFV isolates, a simulated recombinant with 15 times of random recombination event, a genotype I isolate and a genotype II isolate.
